# A novel quantitative real-time PCR with the *GAPDH* reference gene for peste des petits ruminants

**DOI:** 10.17221/123/2023-VETMED

**Published:** 2024-07-29

**Authors:** Yaling Shi, Diangang Han, Jing Li, Lingling Ye, Xincheng Ji, Fuping Nie, Zhigang Song, Chaolin Chen, Jun Ai, Jige Xin

**Affiliations:** ^1^College of Veterinary Medicine, Yunnan Agricultural University, Kunming, P.R. China; ^2^Animal Quarantine Laboratory, Technology Center of Kunming Customs, Kunming, P.R. China; ^3^Research Center for International Inspection and Quarantine Standard and Technical Regulation, General Administration of Customs, Beijing, P.R. China; ^4^Animal and Plant Quarantine Laboratory, Technology Center of Chongqing Customs, Chongqing, P.R. China

**Keywords:** detection method, fluorescence real-time quantitative PCR, internal reference gene, peste des petits ruminants virus

## Abstract

Peste des petits ruminants (PPR) is a serious acute, highly contagious disease caused by the peste des petits ruminants virus (PPRV). This study aims to establish a qRT-PCR assay with an internal amplification control for the rapid and accurate detection of PPRV. The primers and probes for PPRV N were based on the national standard of the diagnostic techniques for PPR of China, and a pair of primers and *Taq*Man probes for the internal reference gene of glyceraldehyde-3-phosphate dehydrogenase (*GAPDH*) was designed. Optimisation of the reaction conditions, specificity, sensitivity and reproducibility tests, and clinical sample detection were conducted. The results showed that the optimal primers and probe concentrations of PPRV were 0.4 μmol/l and 0.4 μmol/l, respectively, and were 0.4 μmol/l and 0.2 μmol/l for the reference gene *GAPDH*, respectively. The established method has no cross-reaction with other viruses. The minimum detection limit was 6.8 copies/μl for PPRV and 190 copies/μl for *GAPDH*. The coefficients of variation (CV%) of PPRV and *GAPDH* were both lower than 2%. The results suggest that the PPRV qRT-PCR method containing internal reference genes has strong specificity, high sensitivity, and good reproducibility. The addition of internal reference genes for the sample quality control improves the accuracy of the detection.

Peste des petits ruminants (PPR) is also known as “goat plague”, “Kata”, “syndrome of stomatitis-pneumoenteritis” or “ovine rinderpest” (Parida et al. 2015). PPR mainly infects small ruminants, especially goats and sheep, which are highly susceptible, and occasionally occurs in wild animals (Diallo et al. 1994). It is a serious virulent, contact infectious disease, and there are no reports of human infection. The morbidity and mortality rates of the disease are as high as 100% and 90%, respectively (Dhar et al. 2002). PPR is classified as a notifiable disease by the World Organization for Animal Health (WOAH), while China has designated it as a Class I animal epidemic. PPR has been reported in most parts of Africa, the Arabian Peninsula, the Near East and the Indian subcontinent (Shaila et al. 1996). It affects and threatens the breeding industry of small ruminants and domestic animals, with an estimated global annual economic loss of 1.2~1.7 billion USD resulting from the mortality, lost production and control costs (Jones et al. 2016). The first PPR record in China was in July 2007 on the Tibetan Plateau (Wang et al. 2009). In response to the outbreak, the government immediately launched a nationwide surveillance programme (Ma et al. 2019). The outbreak of PPR occurred in the Xinjiang Uygur Autonomous Region in November 2013 and quickly spread to 22 provincial-level regions in China in 2014 (Liu et al. 2018). Hunan Province has reported PPR occurrences every year from 2014 to June 2018 (Gao et al. 2019). According to the Ministry of Agriculture and Rural Affairs, on March 24, 2022, the Xinjiang Uygur Autonomous Region Center for Animal Disease Control and Prevention confirmed that a batch of sheep imported from other provinces were infected with PPR. Based on the successful eradication of the rinderpest virus, the Food and Agriculture Organization of the United Nations (FAO) and WOAH are aiming to eradicate PPR globally by 2030 (Dundon et al. 2020).

PPRV is a virus belonging to the genus *Mor-billivirus* of the *Paramyxoviridae* family. The virus particles are encased in nucleocapsids and consist of P phosphoprotein, L microprotein, and N nucleocapsid protein (Bailey et al. 2005). The N protein is encoded by the *N* gene and contains an open reading frame (ORF), which can be compiled into 525 amino acid residues (Diallo et al. 1994). Due to the high abundance of the N protein expression and gene transcription, the N protein is also used as the main target for viral nucleic acid detection. Researchers from various countries have established reverse transcription polymerase chain reaction (RT-PCR) methods targeting the *N* gene for the detection of peste des petits ruminants virus (PPRV). Kwiatek et al. (2010) established a one-step *Taq*Man qRT-PCR method targeting the virus *N* gene to detect four lineages of PPRV. Abera et al. (2014) established SYBR Green I qRT-PCR targeting the *N* gene for PPRV detection.

At present, qRT-PCR is developing rapidly due to its advantages of being fast, simple and suitable for large scale, and it is increasingly used in the diagnosis of animal disease (Bao et al. 2008; Kwiatek et al. 2010). The qRT-PCR method has good specificity, higher accuracy and lower false-positives. However, the qRT-PCR method is prone to generate false-negative results due to errors caused by the procedure of the sample addition, RNA quality of different samples and RNA sample yield in the reverse transcription process (Fleige and Pfaffl 2006). To reduce the error of the test, reference genes are usually selected to correct and standardise the target genes.

In this study, we selected the primers and *Taq*Man probes for the qRT-PCR assay in the national standard of the diagnostic techniques for PPR of China (GB/T 27982-2011) and designed a pair of primers and *Taq*Man probes for the internal reference gene of glyceraldehyde-3-phosphate dehydrogenase (*GAPDH*) based on a comparison of the gene sequences of small ruminants such as sheep, goats and deer. A qRT-PCR method for PPRV containing the reference gene *GAPDH* was established, and the reaction system was optimised to achieve the specificity, sensitivity, and reproducibility of the assay. The addition of internal reference genes for sample quality control improves the accuracy of the detection and can be used for the rapid detection, epidemiological investigation, and epidemic surveillance of PPR. It is helpful to promote the standardisation of qRT-PCR detection methods for PPRV.

## MATERIAL AND METHODS

### Virus and samples

PPRV-positive virus samples (clinically positive samples), bluetongue virus (BTV, type 17 vaccine strain), foot-and-mouth disease virus (FMDV, O/Mya98/XJ/2010 vaccine strain), goat pox virus (GTPV, vaccine strain), Orf virus (ORFV, vaccine strain), epizootic haemorrhagic disease virus (EHDV, vaccine strain), and 92 sheep serum samples were kept in the laboratory of Kunming Customs Technical Center.

### Primer and probe design

The primers and *Taq*Man probes for PPRV qRT-PCR and the primers for ordinary RT-PCR were derived from the national standard of the diagnostic techniques for PPR of China (GB/T 27982-2011), targeting the *N* gene. The design of the primers and probes for the *GAPDH* gene was informed by examining the conserved sequences found in the genome sequences of various small ruminants, including sheep, goats, deer, and other animals. The primers and *Taq*Man probes were synthesised by Sangon Bioengineering (Shanghai) Co., Ltd. (Shanghai, P.R. China) (Table 1).

**Table 1:** The primers and probes sequences of PPRV and *GAPDH*

Name	PCR	Primer name	Sequence
PPRV	RT-qPCR	PPRN8a	CACAGCAGAGGAAGCCAAACT
		PPRN9b	TGTTTTGTGCTGGAGGAAGGA
		PPRN10P	FAM-5'-CTCGGAAATCGCCTCGCAGGCT-3'-TAMRA
	RT-PCR	PPRV-F	TCTCGGAAATCGCCTCACAGACTG
		PPRV-R	CCTCCTCCTGGTCCTCCAGAATCT
*GAPDH*	RT-qPCR	*GAPDH*-F	GGTCACCAGGGCTGCTTTTA
		*GAPDH*-R	CCCGTTCTCAGCCATGTAGT
		*GAPDH*-probe	VIC-5'-TGCCATCAATGACCCCTTCATTGACC-3'-BHQ

*GAPDH* = glyceraldehyde-3-phosphate dehydrogenase; PPRV = peste des petits ruminants virus

### Positive plasmid synthesis

The target sequence of the PPRV *N* gene containing the primers and *Taq*Man probes was synthesised and cloned into a pUC57 vector named puc57-PPRV-*N* by Beijing Tsingke Biotech Co., Ltd (Beijing, P.R. China). The target sequence of *GAPDH* containing the primers and *Taq*Man probes was synthesised and cloned into a pUC57 vector named puc57-*GAPDH* by Beijing Tsingke Biotech Co., Ltd (Beijing, P.R. China).

### *Taq*Man real-time qPCR assay

#### OPTIMISATION OF THE PRIMER AND *TAQ*MAN PROBE CONCENTRATIONS

The upstream and downstream primer concentrations of PPRV and *GAPDH* were set as 0.6 μmol/l, 0.5 μmol/l, 0.4 μmol/l, 0.3 μmol/l and 0.2 μmol/l, respectively, and the *Taq*Man probe concentrations were 0.6 μmol/l, 0.5 μmol/l, 0.4 μmol/l, 0.3 μmol/l, and 0.2 μmol/l. The plasmid puc57-PPRV-*N* was used as a positive template, and puc57-*GAPDH* was used as an internal reference template. The reaction system was set according to the instructions of the HiScript II U+ One Step qRT-PCR Probe Kit (Vazyme, Nanjing, P.R. China): 2 × One Step U+ Mix 10 μl, One Step U+ Enzyme Mix 1 μl, 50 × Rox 0.4 μl, template 1 μl, primers and probes in a corresponding volume, up to 20 μl with H_2_O. The thermal cycling programme consisted of a reverse transcription step at 55 °C for 15 min, 50 cycles with a denaturation step at 95 °C for 10 s, and subsequent annealing and extending at 60 °C for 30 seconds. The concentrations of the primers, *Taq*Man probes, and amplification conditions were optimised to achieve the maximum relative fluorescence units (RFUs) and minimal threshold cycle (Ct).

#### THE ESTABLISHMENT OF STANDARD CURVES

Based on the formula, DNA copies = [6.02 × 10^23^ × plasmid concentrations (ng/μl) × 10^–9^]/(660 × bp of standard plasmids). The numbers of positive copies of puc57-PPRV-*N* and puc57-*GAPDH* were calculated to be 6.8 × 10^10^ copies/μl and 1.9 × 10^10^ copies/μl, respectively. The PPRV-positive plasmid and *GAPDH*-positive plasmid (10^1^~10^8^ dilution ratio) with ddH_2_O in continuous 10-fold gradient dilution were used as templates for the qPCR under optimised reaction conditions. The logarithmic values of the dilution times of the plasmid puc57-PPRV-*N* were used as a positive template and puc57-GAPDH at different concentrations were taken as the X axis, and the mean Ct values were taken as the Y axis to draw standard curves, and the slope and correlation coefficient were calculated.

#### SPECIFICITY TEST

PPRV, BTV, FMDV-O, GTPV, ORFV, and EHDV can infect sheep, goats, deer, and other animals. The mixed- or cross-infection of these pathogens may occur in clinical diagnosis. Therefore, to evaluate the specificity, in this study, nucleic acids were extracted from PPRV, BTV, FMDV-O, GTPV, ORFV, and EHDV and used as the templates. The established qRT-PCR methods containing an internal reference were used for amplification, and the specificity of the method was identified. A negative control group was set up with RNase-free H_2_O.

#### SENSITIVITY TEST

To determine the limit of detection, 10-fold serial dilutions of the plasmid standards were prepared as templates as follows: puc57-PPRV-*N* ranging from 6.8 × 10^0^~6.8 × 10^9^ copies/μl and plasmid puc57-*GAPDH* ranging from 1.9 × 10^0^~1.9 × 10^9^ copies/μl. The sensitivity assay was performed to determine the minimum detection limit of the established method. A negative control group was set with RNase-free H_2_O.

#### REPRODUCIBILITY TEST

To verify the stability of the qPCR assay, the intragroup and intergroup reproducibility of the plasmid with the same concentration was evaluated at least three times in one experiment. The standard deviation of each detected plasmid was calculated separately, and the coefficient of variation was calculated.

Using the established qPCR method with internal parameters, the 10 time consecutively diluted plasmid puc57-PPRV-*N* (6.8 × 10^3^~6.8 × 10^6^ copies/μl) was obtained. The plasmid puc57-*GAPDH* (1.9 × 10^6^~1.9 × 10^9^ copies/μl) was detected by qPCR. Three replicates were set for each gradient dilution ratio as the intragroup repeats and a total of three replicates were performed as the intergroup repeats. A negative control group was set up with RNase-free H_2_O. The mean Ct value, standard deviation (SD), and coefficient of variation (CV) were computed to assess the reproducibility and stability of the established methodology.

#### CLINICAL SAMPLE TESTING

Ninety-two clinical samples of sheep serum stored in our laboratory were extracted with a viral genomic DNA/RNA Nucleic Acid Extraction Kit 4.0 (Tianlong, Xi’an, P.R. China) as the samples to be tested and stored at –80 °C for future use. The qRT-PCR detection method established in this experiment and the common RT-PCR method of the national standard of the diagnostic techniques for PPR of China (GB/T 27982-2011) were used for the detection, and the detection coincidence rates of the two methods were compared and analysed.

The common RT-PCR was performed according to the PrimeScript^TM^ One Step RT-PCR Kit v2 (TaKaRa, Beijing, P.R. China) instructions. Reaction system: PrimeScript 1 Step enzyme mix 2 μl, 2 × 1 step buffer (dye plus) 25 μl, upstream primer 1 μl, downstream 1 μl, template RNA 1 μl, supplement the system to 50 μl using RNase free ddH_2_O. The reaction procedure consisted of a reverse transcription step at 30 min at 50 °C, an enzyme activation step at 94 °C for 2 min, followed by 35 cycles with a denaturation step at 94 °C 30 s, an annealing step at 60 °C 30 s, and an extending step at 72 °C 1 min. After the completion of the reaction, 5 μl of the PCR solution was utilised for agarose gel electrophoresis.

## RESULTS

### Optimisation of the primer and *Taq*Man probe concentrations

**Figure 1:**
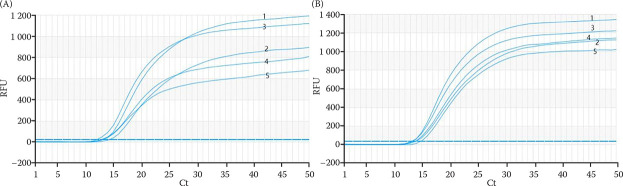
Optimisation of the PPRV primer and probe concentration (A) Optimisation of PPRV primer concentration. 1: 0.6 μmol/l; 2: 0.5 μmol /l; 3: 0.4 μmol /l; 4: 0.3 μmol/l; 5: 0.2 μmol/l; (B) Optimisation of PPRV probe concentration. 1: 0.6 μmol/l; 2: 0.5 μmol /l; 3: 0.4 μmol /l; 4: 0.3 μmol/l; 5: 0.2 μmol/l

**Figure 2:**
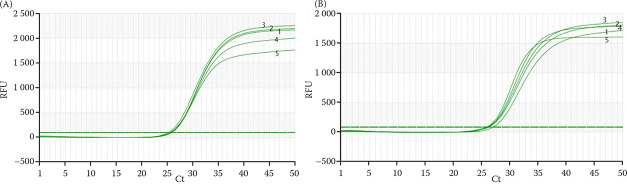
Optimisation of the *GAPDH* primer and probe concentration (A) Optimisation of *GAPDH* primer concentration. 1: 0.6 μmol /l; 2: 0.5 μmol /l; 3: 0.4 μmol /l; 4: 0.3 μmol/l; 5: 0.2 μmol/l; (B) Optimisation of *GAPDH* probe concentration. 1: 0.6 μmol /l; 2: 0.5 μmol /l; 3: 0.4 μmol /l; 4: 0.3 μmol/l; 5: 0.2 μmol/l

The primer and *Taq*Man probe concentration were optimised using plasmid puc57-PPRV-*N* diluents (6.8 × 10^10^ copies/μl) and plasmid puc57-*GAPDH* diluents (1.9 × 10^10^ copies/μl) as the templates, respectively. The optimal primer and *Taq*Man probe concentration were selected based on the shape of the amplification curve, Ct value and fluorescence intensity. After optimisation, the optimal concentration of the upstream and downstream primers of PPRV and the *Taq*Man probe was 0.4 μmol/l, the optimal concentration of the upstream and downstream primers of *GAPDH* was 0.4 μmol/l, and the *Taq*Man probe concentration was 0.2 μmol/l (Table 2, Table 3, Figure 1, Figure 2).

**Table 2:** Optimisation of the PPRV primer and probe concentration

Primer quantity (μl)	Primer concentration (μmol/l)	Ct value
1.2	0.6	13.36
1.0	0.5	14.47
0.8	0.4	12.18
0.6	0.3	12.93
0.4	0.2	13.12
*Taq*Man probe quantity(μl)	*Taq*Man probe concentration (μmol/l)	Ct value
1.2	0.6	13.35
1.0	0.5	14.01
0.8	0.4	13.16
0.6	0.3	13.79
0.4	0.2	14.82

Ct = threshold cycle; PPRV = peste des petits ruminants virus

**Table 3:** Optimisation of the *GAPDH* primer concentration

Primer quantity (μl)	Primer concentration (μmol/l)	Ct value
1.2	0.6	26.57
1.0	0.5	26.41
0.8	0.4	25.75
0.6	0.3	26.08
0.4	0.2	26.41
*Taq*Man probe quantity (μl)	*Taq*Man probe concentration (μmol/l)	Ct value
1.2	0.6	27.54
1.0	0.5	26.75
0.8	0.4	26.53
0.6	0.3	26.46
0.4	0.2	26.32

Ct = threshold cycle; *GAPDH* = glyceraldehyde-3-phosphate dehydrogenase

### The establishment of a standard curve

**Figure 3:**
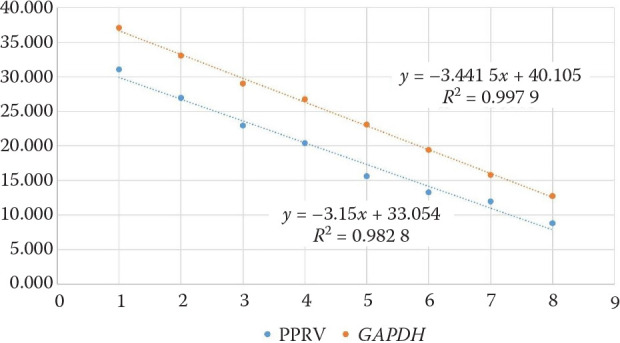
Figure 3. The standard curves

Eight standards of the continuous gradient (10^1^~10^8^ dilution) were selected as the X-axis, and the obtained Ct value was the Y-axis to draw the standard curve (Figure 3). The equation of the PPRV standard curve was *y *= –3.15*x* + 33.054, with a correlation coefficient *R*^2 ^= 0.982 8, and the standard curve equation of *GAPDH* was *y* = –3.441 5*x* + 40.105, with a correlation coefficient *R*^2 ^= 0.997 9, indicating that the gradient dilution template showed a good linear relationship with the Ct value.

### Specificity test

**Figure 4:**
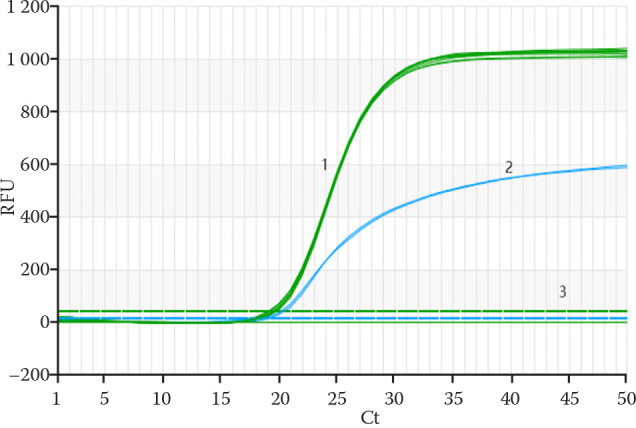
The specificity tests 1: Positive plasmid puc57-*GAPDH*; 2: PPRV positive virus samples; 3: BTV, FMDV, GTPV, ORFV, EHDV and negative controls BTV = bluetongue virus; EHDV = epizootic haemorrhagic disease virus; FMDV = foot-and-mouth disease virus; *GAPDH* = glyceraldehyde-3-phosphate dehydrogenase; GTPV = goat pox virus; ORFV = Orf virus; PPRV = peste des petits ruminants virus

The established qRT-PCR method was used for the detection of PPRV, BTV, FMDV-O, GTPV, ORFV, and EHDV. The results showed that all the internal *GAPDH* parameters of the detected samples were positive (green). Only the PPRV-positive virus samples showed amplification curves (blue), while the other viruses and negative controls showed no amplification curves and no fluorescence signal, indicating that the established method had good specificity (Figure 4).

### Sensitivity test

**Figure 5:**
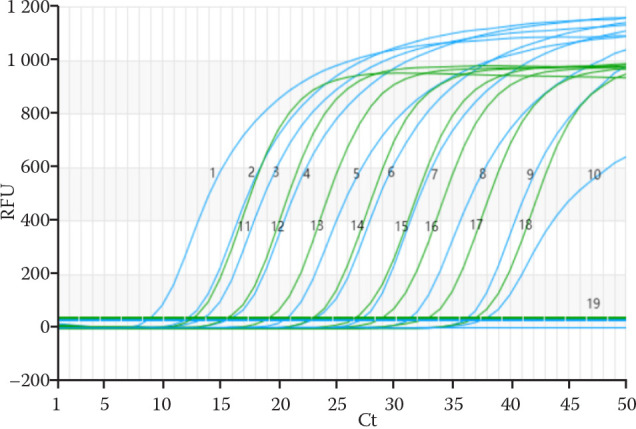
The sensitivity tests 1~10: the positive plasmid puc57-PPRV-*N* from 6.8 × 10^9^ copies/μl~6.8 copies/μl (blue lines); 11~18: the posi-tive plasmid puc57-*GAPDH* from 1.9 × 10^9^ copies/μl~1.9 × 10^2^ copies/μl (green lines); 19: negative controls (green lines)

The qPCR detection was performed on the 10 time consecutively diluted positive plasmid puc57-PPRV-*N* (6.8~6.8 × 10^9^ copies/μl) and positive plasmid puc57-*GAPDH* (1.9~1.9 × 10^9^ copies/μl). The results are shown in Figure 5. The minimum detectable limit of positive plasmid puc57-PPRV-*N* was 6.8 copies/μl and that of positive plasmid puc57-*GAPDH* was 190 copies/μl, indicating that the established method had good sensitivity.

### Reproducibility test

The experimental results showed that the standard deviation of the positive plasmid puc57-PPRV-*N* (6.8 × 10^3^~6.8 × 10^6^ copies/μl) within the group was 0.02~0.36, the coefficient of variation was 0.12%~1.64%, and the standard deviation of the intergroup detection was 0.07~0.47. The coefficient of variation ranged from 0.25% to 1.86% (Table 4). The standard deviation of the positive plasmid puc57-*GAPDH* (1.9 × 10^6^~1.9 × 10^9^ copies/μl) was 0.01~0.20, the coefficient of variation was 0.06%~1.05%, and the standard deviation of the intergroup detection was 0.03~0.19. The coefficient of variation is 0.13%~1.16%, indicating that the proposed method has good stability and reproducibility (Table 5).

**Table 4:** The reproducibility results for the qPCR assay against PPRV

Copy number (copies/μl)	Intragroup repetition				Intergroup repetition		
	means	SD	CV (%)		means	SD	CV (%)
6.8 × 10^6^	18.29	0.021 6	0.12		18.38	0.095 2	0.52
6.8 × 10^5^	22.00	0.361 2	1.64		21.72	0.242 1	1.11
6.8 × 10^4^	25.84	0.291 3	1.13		25.20	0.468 8	1.86
6.8 × 10^3^	28.70	0.189 1	0.66		28.63	0.073 2	0.26

CV = coefficient of variation; PPRV = peste des petits ruminants virus; SD = standard deviation

**Table 5:** The reproducibility results for the qPCR assay against *GAPDH*

Copy number (copies/μl)	Intragroup repetition				Intergroup repetition		
	means	SD	CV (%)		means	SD	CV (%)
1.9 × 10^9^	13.45	0.075 9	0.56		13.37	0.129 1	0.97
1.9 × 10^8^	16.80	0.176 3	1.05		16.69	0.192 9	1.16
1.9 × 10^7^	20.17	0.012 5	0.06		20.15	0.026 0	0.13
1.9 × 10^6^	23.30	0.200 4	0.86		23.35	0.075 4	0.32

CV = coefficient of variation; *GAPDH* = glyceraldehyde-3-phosphate dehydrogenase; SD = standard deviation

### Clinical sample testing

**Figure 6:**
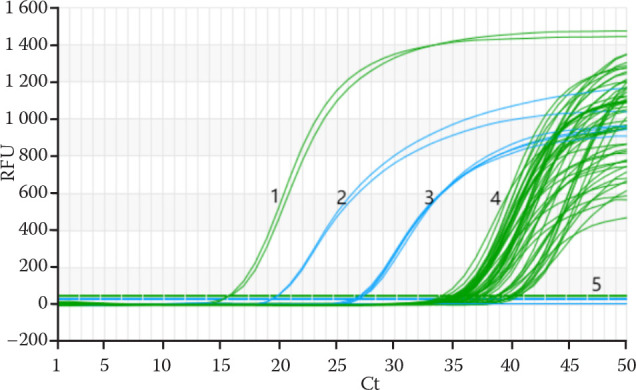
The clinical sample detection by RT-qPCR 1: Positive control of puc57-*GAPDH* plasmid; 2: Positive control of puc57-PPRV-*N* plasmid; 3: PPRV positive samples; 4: GAPDH positive samples; 5: negative controls

**Figure 7:**
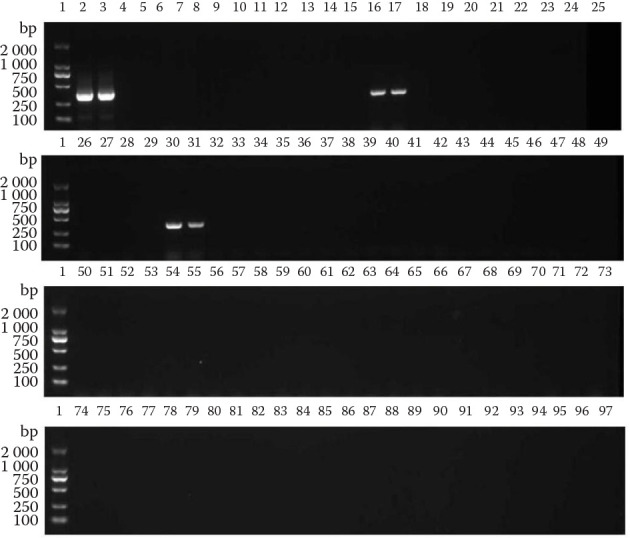
The clinical sample detection by RT-PCR 1: 2 000 maker; 2~3: Positive plasmid control of puc57-PPRV-*N* control; 4~5: negative control; 6~97: sheep serum clinical samples, of which 16, 17, 30, 31 tested positive for PPRV, the rest of the samples tested negative

Ninety-two clinical samples of the sheep serum stored in the laboratory were detected by the qRT-PCR established in this study and the RT-PCR method in the national standard of the diagnostic techniques for PPR of China (GB/T 27982-2011), and the coincidence rate of the detection results of the two methods was analysed. The results showed that the clinical specimens were all positive for the *GAPDH* gene amplification by the qRT-PCR method established in this study, and the experimental results were valid. The detection results of the PPRV qRT-PCR (Figure 6) and the national standard ordinary RT-PCR (Figure 7) were both positive in four cases and negative in 88 cases, and the coincidence rate of the two detection methods was 100%. The results indicated that the qRT-PCR method containing the reference PPRV was established successfully.

## DISCUSSION

PPR is a notifiable animal disease of the World Organization for Animal Health (WOAH). Currently, qRT-PCR has become a common method to detect PPRV. qRT-PCR can be used to detect animal and human viruses, such as identifying genotypes and quantifying viral pathogens, with the advantages of accurate quantification, strong specificity and high sensitivity. The PCR detection method of PPRV has been studied by scholars. Balamurugan et al. (2012), Bao et al. (2008), Kwiatek et al. (2010) and Abera et al. (2014), who all established a qRT-PCR for PPRV. These qRT-PCR methods are faster and more sensitive than the conventional RT-PCR method and can be used to detect PPRV nucleic acids in clinical samples of sheep and goats with suspected PPR. However, in qRT-PCR, to obtain accurate results, internal reference genes often need to be introduced to correct for differences in the initial template purity and concentration of the different samples and reverse transcription amplification efficiency (Park et al. 2013).

*GAPDH* is the most commonly used internal reference gene, which is a key enzyme in the glycolysis pathway and closely related to adenosine triphosphate (ATP) synthesis. It exists widely in many organisms and has a highly conserved species sequence (Kubo et al. 2011). Wang et al. (2022) used qRT-PCR to screen and validate reference genes in bovine skeletal muscle-derived satellite cells and the results showed that *GAPDH* was the most stable reference gene during the proliferation of bovine pre-primary adipocytes and bovine skeletal muscle-derived satellite cells *in vitro*. Peletto et al. (2011) used real-time qPCR to detect the quantitative expression of the internal reference genes in the whole blood of sheep, and the results showed that *GAPDH* was the most stable in the whole blood under disease conditions. de Cassia-Pires et al. (2017) established multiplex PCR as a diagnostic tool for Leishmania parasite kDNA and mammalian host *GAPDH* housekeeping genes. In these studies, *GAPDH* is often used as an internal reference gene for the disease detection and gene expression. However, *GAPDH* has not been used to establish a qRT-PCR detection method for PPRV.

Therefore, a qRT-PCR method containing the internal reference *GAPDH* gene was established in this study. Based on the addition of internal parameters, the low detection limit of the qRT-PCR method established by us was 6.8 copies/μl, which was better than the 8.1 copies/μl of the qRT-PCR method established by Bao et al. (2008). The intragroup and intergroup coefficients of variation (CV%) of PPRV were 0.06%~1.05% and 0.13%~1.16%, respectively, which were smaller than the intragroup and intergroup coefficients of variation of 0.32%~2.31% and 0.71~5.32% established by Abera et al. (2014). Therefore, the addition of the internal reference *GAPDH* had no effect on the sensitivity of the established method, and after optimisation, its sensitivity and reproducibility were higher than those of the other published qRT-PCR methods. qRT-PCR was performed on 92 clinical samples of sheep serum, and the results were consistent with the common RT-PCR method in the national standard of the diagnostic techniques for PPR of China (GB/T 27982-2011). Esonu et al. (2022) described that 14 articles (37.8%) reported seropositive rates in small ruminants, ranging from 0.0% to 77.5% when more than 10 animals were sampled, which is consistent with our test results.

The qRT-PCR method established in this study for the simultaneous detection of PPRV and the internal reference gene *GAPDH* exhibits sensitivity, specificity, and reproducibility. Incorporating an internal reference gene effectively ensures the detection accuracy and minimises any false-negative results. This method is suitable for the rapid peste des petits ruminants detection with a large sample size, making it applicable to epidemiological investigations and epidemic monitoring. Furthermore, it serves as a valuable reference for standardising qRT-PCR methods in diagnosing peste des petits ruminants disease.
